# Orthodontic treatment outcomes obtained by application of a finishing protocol

**DOI:** 10.1590/2177-6709.21.2.088-094.oar

**Published:** 2016

**Authors:** Alvaro Carvajal-Flórez, Diana María Barbosa-Lis, Oscar Arturo Zapata-Noreña, Julissa Andrea Marín-Velásquez, Sergio Andrés Afanador-Bayona

**Affiliations:** 1 Assistant Professor, Universidad de Antioquia, Orthodontics Graduate Program, Medellín, Colombia.; 2 Associated Professor, Universidad de Antioquia, Orthodontics and Maxillary orthopedics program, Antioquia, Colombia.; 3 Full Professor, Universidad de Antioquia, Orthodontics and Maxillary Orthopedics Program, Medellín, Colombia.; 4 Resident, Universidad de Antioquia, Orthodontics Graduate Program, School of Dentistry, Antioquia, Colombia.; 5 Resident, Universidad de Antioquia, Orthodontics Graduate Program, School of Dentistry, Antioquia, Colombia.

**Keywords:** Treatment outcomes, Index of Orthodontic Treatment Need, Malocclusion, Clinical protocols.

## Abstract

**Objective:**

To evaluate the results of a finishing protocol implemented in patients treated in the Orthodontics graduate program at Universidad de Antioquia. Evaluation was carried out by means of the criteria set by the Objective Grading System (OGS) of the American Board of Orthodontics (ABO).

**Methods:**

Cast models and panoramic radiographs of 34 patients were evaluated. The intervention group (IG) consisted of 17 patients (19.88 ± 4.4 years old) treated under a finishing protocol. This protocol included training in finishing, application of a finishing guide, brackets repositioning and patient's follow-up. Results of the IG were compared to a control group of 17 patients (21.88 ± 7.0 years old) selected by stratified randomization without finishing intervention (CG).

**Results:**

The scores for both CG and IG were 38.00 ± 9.0 and 31.41 ± 9.6 (*p* = 0.048), respectively. The score improved significantly in the IG group, mainly regarding marginal ridges (CG: 5.59 ± 2.2; IG: 3.65 ± 1.8) (*p* = 0.009) and root angulation (CG: 7.59 ± 2.8; IG: 4.88 ± 2.6) (*p* = 0.007). Criteria that did not improve, but had the highest scores were: alignment (CG: 6.35 ± 2.7; IG: 6.82 ± 2.8) (*p* = 0.62) and buccolingual inclination (CG: 3.6 ± 5.88; IG: 5.29 ± 3.9) (*p* = 0.65).

**Conclusions:**

Standardization and implementation of a finishing protocol contributed to improve clinical performance in the Orthodontics graduate program, as expressed by occlusal outcomes. Greater emphasis should be given on the finishing phase to achieve lower scores in the ABO grading system.

## RESUMO

**Objetivo:**

avaliar os resultados da implementação de um protocolo de finalização em pacientes tratados no programa de pós-graduação em Ortodontia da Universidad de Antioquia. A avaliação foi conduzida utilizando-se os critérios definidos pelo *Objective Grading System* (OGS) do *American Board of Orthodontics* (ABO).

**Métodos:**

modelos de gesso e radiografias panorâmicas de 34 pacientes foram avaliados. O grupo experimental (GE) consistiu de 17 pacientes (idade média = 19,88 ± 4,4 anos) submetidos a um protocolo de finalização que incluiu o treinamento para a etapa de finalização, a aplicação de um guia de finalização, o reposicionamento de braquetes e o acompanhamento dos pacientes. Os resultados obtidos para o GE foram comparados aos resultados de um grupo controle (GC), não submetido à etapa de finalização, composto por 17 pacientes (idade média = 21,88 ± 7,0 anos) selecionados por um método de amostragem aleatória estratificada.

**Resultados:**

os escores do GC e do GE foram de 38,00 ± 9,0 e 31,41 ± 9,6 (*p* = 0,048), respectivamente. Houve melhora significativa no escore do grupo GE, principalmente com relação às cristas marginais (GC = 5,59 ± 2,2; GE = 3,65 ± 1,8) (*p* = 0,009) e à angulação da raiz (GC = 7,59 ± 2,8; GE = 4,88 ± 2,6) (*p* = 0,007). Os critérios que não apresentaram melhora, mas obtiveram os escores mais altos, foram: alinhamento (GC = 6,35 ± 2,7; GE = 6,82 ± 2,8) (*p* = 0,62) e vestibularização (GC = 3,6 ± 5,88; GE = 5,29 ± 3,9) (*p* = 0,65).

**Conclusões:**

a implementação de um protocolo padronizado de finalização contribuiu para melhorar o desempenho clínico dos alunos em um programa de pós-graduação em Ortodontia, conforme demonstram os resultados oclusais. Maior ênfase deveria ser dada à fase de finalização, para se obter escores mais baixos no OGS do ABO.

## INTRODUCTION

Any orthodontic treatment is an effort to obtain the best possible esthetic and functional occlusion for the patient. To initiate the finishing stage of treatment, teeth must be aligned, extraction spaces must be closed and posterior teeth must have a Class I occlusal relationship. The finishing stage includes: obtain parallel roots, adjust the position of individual teeth to correct mesiodistal and buccolingual inclinations, and level marginal ridges by correction of bracket positions in order to obtain an ideal occlusion.^1^

The outcomes of finished orthodontic treatments are assessed by employing different criteria to express the quality of final results. In 1998, the American Board of Orthodontics (ABO) introduced the Objective Grading System (OGS) or Cast/Radiographic Evaluation (CRE), a standard method to evaluate finished cases considering eight criteria (alignment, marginal ridges, buccolingual inclination, occlusal relationships, occlusal contacts, overjet, interproximal contacts and root angulation) in dental casts and panoramic radiographs.^3^

For the graduate program of Orthodontics at the Universidad de Antioquia, it is important to have a tool to evaluate clinical processes and outcomes by means of a system that provides reliable quantitative information, comparable to other similar institutions. In the School of Dentistry of Universidad de Antioquia, during the years of 2010-2011,^4^ a total of 40 finished patients were evaluated by the Objective Grading System (OGS). The average standard deviation score was: 31.7 ± 8.4 points. This value is not very high, considering that scores over 30 points are qualified as less than acceptable; however, it is within the averages previously reported by other university orthodontic programs.^5^ ^,^ ^6^ ^,^ ^7^ ^,^ ^8^ In the Okayama University, an evaluation of 72 patients carried out by the OGS index was compared to results of 54 patients from the Indiana University. The scores were: 33.6 ± 13.6 and 32.8 ± 10.3, respectively.^7^ In the Universidad de Puerto Rico, during the years of 2007-2008, a total of 64 patients were evaluated by OGS, obtaining a score of 32.17 ± 13.0.^8^ The records of 521 finished patients in the Indiana University had OGS scores of 32.43 ± 10.5, 33.09 ± 10.2 and 37.83 ± 9.5, respectively, for the years of 1998, 1999 and 2000.^6^ The Universidad de Manizales, Colombia, reported OGS values of 30.94 ± 11.0 for a sample of 31 patients, in a study in which 54.8% of cases were approved according to the ABO criteria.^9^ At the University of Illinois, 92 finished cases were compared to 32 cases treated by five orthodontists certified by the ABO. The OGS score was 45.54 ± 18.3 for university patients, significantly higher than the score obtained in private practice (33.88 ± 9.6).^5^

At the Indiana University, based on the results measured by OGS criteria and the pattern of deficiencies detected, a series of curricular changes was introduced with a view to improving the efficiency of treatment. Following the introduction of those changes, the Indiana University reported OGS indexes of 28.66 ± 13.0 in 2001, 24.97 ± 9.4 in 2002, and 22.42 ± 10.0 in 2003.^10^

The University of Detroit, considering that the mean OGS scores were 31.16 and 34.79, for 2003 and 2004, respectively, introduced curricular modifications to generate better follow-up of cases, in addition to reducing patients' referrals. Due to these modifications, the mean OGS changed from 28.55 ± 1.7 in 2005 to 22.11 ± 1.0 in 2007.^11^

The objective of the present study was to evaluate the results obtained after the implementation of a finishing protocol for patients treated in the clinics of the Graduate Orthodontics program at Universidad de Antioquia, Colombia.

## MATERIAL AND METHODS

The study evaluated conventional dental casts and digital panoramic radiographs of 34 patients. The sample was taken by convenience and distributed into two groups: intervention group (IG) (n = 17), selected according to inclusion and exclusion criteria; and a control group (CG) (n = 17), selected by stratified sampling of treated patients not following the finishing protocol. The inclusion criteria to select IG patients were:

» Patients initiating the finishing stage of treatment.
» Complete diagnostic records.
» No surgical maxillofacial, periodontal or prosthetic treatment required.
» Patients that completed treatment according to the concept of the clinician in charge.
» Acceptance to participate in the study by signing an informed consent form.

The exclusion criteria were patients who did not adhere to the protocol, and patients who decided to remove the appliances at their own willing.

Due to ethical restriction for a random prospective distribution of patients into two groups when a benefit from the intervention is theoretically expected, the Ethics Board of the School of Dentistry suggested to apply the protocol to all patients treated between 2014-2015 (IG), and use a historical group of patients finished between 2010-2011, when the finishing protocol was not applied, as a control group (CG). The finishing protocol included the following activities:

» Information and training of students and professors of the Orthodontics graduate program about occlusal characteristics evaluated by the OGS as well as about specific deficiencies found in a previous study.^4^
» Analysis of dental cast and panoramic radiographs as defined by the ABO-OGS (alignment, marginal ridges, bucco-lingual inclination, occlusal relationships, occlusal contacts, overjet, interproximal contacts and root angulation) using the protocol named *UdeA Finishing Guide* (that also includes midline evaluation, overbite, smile arch and smile line).
» Strict supervision of graduate students by professors to ensure compliance in using the *UdeA Finishing Guide*.
» Correction of discrepancies, giving priority to early repositioning of brackets over arch-wire bending.
» Bimonthly checking of the protocol implementation by the research group.

Following the application of corrective actions indicated by the *UdeA finishing guide*, fixed appliances were removed and final dental casts as well as radiographs were taken at the diagnostic center IMAX^TM^ (Medellín, Colombia) by means of standardized procedures. Data and registers of the control group were also taken at this center under the same technical parameters.^4^

The assessment of seven occlusal parameters^4,12,13^ established by the OGS in dental casts was digitally obtained by means of Ortho Insight 3D scanner (Motion View Software, LLC, Chattanooga TN, USA). The system was calibrated to provide a confidentiality of 95%. Marginal ridge discrepancy was manually evaluated with an instrument that fulfills ABO specifications,^3^ validated and certified by Mebi Metrología (Metrología Biomédica, Medellín, Colombia) at 95% of accuracy. According to the ABO, evaluation of root parallelism was manually assessed in the panoramic radiograph. The final OGS value was obtained by adding the results of dental cast and radiographic measurements. The method described by Barbosa et al^4^ was followed in order to assess malocclusion complexity. It uses information from the clinical records according to affected planes: transversal, vertical, sagittal, alignment and others (the presence of additional findings, such as supernumerary teeth, dental transpositions and/or unerupted teeth).

The examiners were trained to obtain intra- and interexaminer Kappa coefficients higher than 0.80 and were blinded with respect to patients' groups.

Statistical analysis of data was performed by means of SPSS v.19 software (SPSS Inc., Chicago, IL, USA). Univariate description of quantitative variables included mean and standard deviation calculations, and qualitative results were described as frequency distributions. Multivariate analysis was used to estimate the influence of each independent variable over the outcome measured by OGS. The level of significance was *p* < 0.05. This investigation is registered in ClinicalTrials.gov: NCT02290158. The results of the investigation are presented following CONSORT indications.^14^

### Ethical issues

According to the WMA Declaration of Helsinki and Resolution 8430 of 1993 from the Colombian Ministry of Health, this study was classified as having a risk higher than the minimum, since patients were submitted to radiographic exposure. This exposure is not additional to that caused by conventional orthodontic diagnosis. The Ethics Committee of the School of Dentistry, Universidad de Antioquia, authorized this research project as documented by Act 10 of 2013. Patients signed an informed consent form.

## RESULTS 

The demographic characteristics of the sample of 34 patients are summarized in Table 1. The IG group included seven men and ten women aged 19.88 ± 4.41 years; whereas the CG group included eight men and nine women aged 21.88 ± 7.09 years. Treatment time was 59.29 ± 28.98 months for IG and 53.59 ± 13.49 months for CG. The difference between groups regarding sex (*p* = 0.73), age (*p* = 0.33) or treatment time (*p* = 0.46) was not significant. The mean time between the implementation of the protocol and the end of treatment was 11.41 ± 4.97 months.

**Table 1:** Sex, age and time of treatment for each group.

Variables	CG	IG	*p* value
	mean ± SD	mean ± SD	
Sex	7 men	8 men	0.73
	10 women	9 women	Chi-square
Age (years)	21.88 ± 7.09	19.88 ± 4.41	0.33
			Student's t-test
Treatment time (Months)	53.59 ± 13.49	59.29 ± 28.98	0.46
			Student's t-test

* Statistically significant difference (*p* < 0.05).

**Figure 1:**
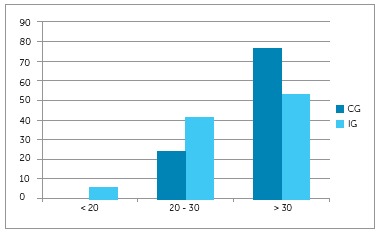
Percentage of patients classified by OGS score for each group.

**Figure 2:**
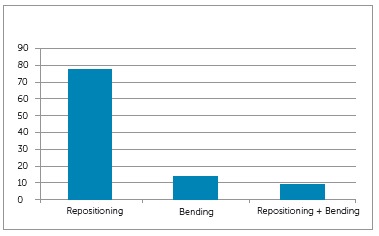
Percentage of intervention strategies used during implementation of the finishing protocol.

The analysis of OGS values between groups found significant differences for marginal ridges (*p* = 0.009), root angulation (*p* = 0.007) and total OGS (*p* = 0.048), as summarized in Table 2. In Table 3, the total OGS results for Grade of Commitment between groups are described. The difference was significant (*p* = 0.033) when highly compromised patients were compared. The percentage of subjects classified by OGS score is shown in Figure 1. Additionally, Figure 2 shows the percentage of strategies implemented.

**Table 2:** OGS score (mean± S.D.) by components and groups.

Variables	CG (n = 17)	%	IG (n = 17)	%	*p* value
Alignment	6.35 ± 2.71	16.71	6.82 ± 2.87	21.71	0.62
Marginal ridges	5.59 ± 2.21	14.71	3.65 ± 1.83	11.62	0.009*
Buccolingual inclination	5.88 ± 3.68	15.47	5.29 ± 3.93	16.84	0.65
Occlusal relationships	6.18 ± 2.81	16.26	4.94 ± 3.23	15.72	0.24
Occlusal contacts	2.35 ± 2.31	6.18	2.47 ± 3.08	7.86	0.90
Overjet	4.00 ± 3.50	10.52	3.29 ± 3.25	10.47	0.54
Interproximal contacts	0.06 ± 0.24	0.15	0.06 ± 0.25	0.00	0.96
Root angulation	7.59 ± 2.80	19.97	4.88 ± 2.69	15.53	0.007*
Total	38.00 ± 9.01	100	31.41 ± 9.67	100	0.048*

* Statistically significant difference (*p* < 0.05).

**Table 3:** OGS score according to occlusion complexity by group.

Occlusion complexity	CG mean ± SD	IG mean ± SD	*p* value
LC (Low complexity) (up to two planes)	28.00 ± 2.00	27.00 ± 13.45	0.905
C (Complex) (three planes)	37.71 ± 7.04	33.43 ± 10.56	0.389
HC (High complexity) (more than three planes)	42.57 ± 9.55	31.29 ± 7.93	0.033*

* Statistically significant difference (*p* < 0.05).

## DISCUSSION

This study evaluated the outcomes obtained when a standardized finishing protocol was applied to patients treated at the Orthodontics clinics of Universidad de Antioquia during a period of 16 months, from 2014 to 2015. The results were evaluated according to ABO-OGS criteria and compared with the results of a control group finished in 2010-2011, when the protocol was not applied. The OGS scores are 38 ± 9.01 and 31.41 ± 9.67, for CG and IG, respectively. This difference is statistically significant (Table 2). Therefore, the application of the protocol contributed to the reduction in OGS mean value; however, the total OGS score obtained is still high, as the ABO states that scores higher than 30 points are not acceptable.^14^ The results of the present study are similar to values reported by the Orthodontics program of the Indiana University, which revealed some improvement after the introduction of corrective strategies. The percentage of patients who finished with OGS values less than 30 points changed from 39.7% to 76.6%.^10^ The present study shows that nearly half of patients treated according to the finishing protocol achieved better outcomes according to international standards (Fig 1).

When specific components of the OGS, showing significant differences between groups, were compared, it was observed that marginal ridge and root angulation are better for IG than for CG (Table 2). In other words, the OGS component score was lower in IG compared to CG.

Poling^15^ recommends that, four to seven months before having orthodontic appliances removed, the pa tient should be evaluated using a check-list ,in order to attain excellent outcomes. The finishing protocol applied to the IG group was designed by the authors, taking into account the ABO-OGS criteria and the evaluation of panoramic radiographs as well as cast models of patients at the beginning of the finishing stage. Before the introduction of the ABO-OGS system, orthodontists did not have a tool for objective qualification of cases at the end of treatment, and that was one of the reasons for not being approved by the ABO, since final occlusion was inadequate.^10^ The control group was treated without having OGS parameters checked up; in addition, casts and panoramic radiographs were frequently not taken into account to finish the case. The case used to be considered as finished when only professors' subjective criteria were followed. The results of the present study clearly in dicate the importance of knowledge of ideal occlusion as well as the relevance of the evaluation of clinical records taken for finishing stage. 

The finishing guide appears to be necessary to identify and correct mistakes in dental and root positions, so as to reduce the final OGS score.

The protocol implemented in the IG group, which is similar to that established by Knierim et al,^15^ suggests that, when it becomes necessary to correct tooth position, it is better to reposition the bracket instead of introducing new arch bendings.^10^ Other authors consider that bracket repositioning might not be correct, perpetuating the error previously identified. When the *UdeA Finishing Guide* was evaluated, it was observed that the most frequent strategy to correct dental position was repositioning of brackets and tubes (Fig 2). However, some components evaluated by the OGS did not significantly improve. This finding suggests that some manipulations performed to correct mistakes previously detected might not be adequate due to imprecision in the new location of the bracket. It is also possible that, in some cases, the need for correction was not detected. It was also observed that the intervention demanded for the same tooth was, in some situations, a combination of bracket repositioning and new arch bending, thereby suggesting that the strategy of repositioning may not be sufficient to correct one or more errors in dental position.

The Discrepancy Index (DI) was developed by the ABO as an objective tool to describe complexity of treatment, based upon observations and measurements taken in pretreatment dental casts, cephalic and panoramic radiographs.^16^ The lack of initial registers, standardized for both groups of patients, precluded the application of DI to analyze its relationship with OGS. Instead, the grade of commitment described by Barbosa et al^4^ was assessed, which appears to be related to OGS results. This indicates that patients initiating orthodontic treatment with a high level of complexity tend to finish it with higher OGS grades, and vice versa. The present study shows that highly compromised patients finished treatment with a higher OGS score. However, the correlation between OGS and grade of commitment was not statistically significant, either due to sample size or lack of precision of the instrument used to evaluate patients' commitment. 

Pinskaya et al^6^ reported that longer treatment time is related to worse finishing results. In the present study, there is no correlation between duration of treatment and OGS scores in any group. This finding agrees with the study by Campbell et al.^17^ The time of the finishing stage of treatment could not be compared between the two groups due to lack of appropriate date records for the control group. However, total treatment time was not significantly different between the two groups. Therefore, it is apparent that the application of the finishing protocol improved clinical results without increasing treatment time. 

One possible limitation of this study is that the professors who performed the supervision of the orthodontic treatment without finishing protocol four years before may have improved their clinical skills since then, and this aspect could have affected the final scores in the experimental group. However, one of the objectives of this trial was to improve the knowledge and skills of professors. Apart from this aspect, the improvement of the score should be attributed to the application of the whole protocol as such, which had a training effect on all participants.

## CONCLUSIONS

The implementation of a standardized protocol for the finishing stage of orthodontic treatments in the graduate clinics of Orthodontics of Universidad de Antioquia improved the occlusal outcomes of treatment. More emphasis must be given during the finishing stage to improve aspects that still present high scores.
